# Supplementary material for: Empirical methods for controlling false positives and estimating confidence in ChIP-Seq peaks
Source: BMC Bioinformatics. 2008 Dec 5;9:523. doi: 10.1186/1471-2105-9-523 (PMC2628906; doi:10.1186/1471-2105-9-523)
Supplement: Additional File 1 — A variety of html documents from the USeq web site detailing the available applications, their best usage, output file type descriptions, command line menus, etc. [file 1471-2105-9-523-S1.zip › USeqUserGuides/JavaDoc.zip!/JavaDoc/util/bio/parsers/gff/GffFeature.html]

GffFeature


|  |  |  |
| --- | --- | --- |
| **Overview** \| **Package** \| **Class** \| **Use** \| **Tree** \| **Deprecated** \| **Index** \| **Help** | |  |
| **PREV CLASS**   **NEXT CLASS** | **FRAMES**    **NO FRAMES**     **All Classes** |  |
| SUMMARY: NESTED \| FIELD \| CONSTR \| METHOD | DETAIL: FIELD \| CONSTR \| METHOD |  |


---


## util.bio.parsers.gff Class GffFeature

```
java.lang.Object
  util.bio.parsers.gff.GffFeature
```

---

``` public class GffFeature extends java.lang.Object ```

A container for each of the values in a GFF 2 line.
tabtabtabtabtabtab
tabtab[space delimited key=value; key=value; attributes]
Each param is split into a String[] on \t
Attributes are further processed splitting on ;\s into another String[] each
of theses Strings are then split into key value pairs on an = or \s and stuffed
into a LinkedHashMap
Note Stop = End +1, needed to convert to real coordinates!

---

**Constructor Summary**

`GffFeature(java.lang.String unParsedGffLine)`


| **Method Summary** | |
| --- | --- |
| `java.util.LinkedHashMap` | `getAttsHash()` |
| `java.lang.String` | `getAttsString()` |
| `int` | `getEnd()` |
| `java.lang.String` | `getFeature()` |
| `java.lang.String` | `getGffLine()` |
| `double` | `getScore()` |
| `java.lang.String` | `getSeqName()` |
| `java.lang.String` | `getSource()` |
| `int` | `getStart()` |
| `java.lang.String` | `getStrand()` |
| `boolean` | `isGffGood()` |
| `static double` | `parseStringToDouble(java.lang.String text)` |
| `java.lang.String` | `toString()` |

**Methods inherited from class java.lang.Object**

`equals, getClass, hashCode, notify, notifyAll, wait, wait, wait`

**Constructor Detail**

### GffFeature

```
public GffFeature(java.lang.String unParsedGffLine)
```


**Method Detail**

### toString

```
public java.lang.String toString()
```

:   **Overrides:**: `toString` in class `java.lang.Object`

---


### parseStringToDouble

```
public static double parseStringToDouble(java.lang.String text)
```

---


### getGffLine

```
public java.lang.String getGffLine()
```

---


### getSeqName

```
public java.lang.String getSeqName()
```

---


### getSource

```
public java.lang.String getSource()
```

---


### getFeature

```
public java.lang.String getFeature()
```

---


### getStart

```
public int getStart()
```

---


### getEnd

```
public int getEnd()
```

---


### getScore

```
public double getScore()
```

---


### getStrand

```
public java.lang.String getStrand()
```

---


### getAttsHash

```
public java.util.LinkedHashMap getAttsHash()
```

---


### getAttsString

```
public java.lang.String getAttsString()
```

---


### isGffGood

```
public boolean isGffGood()
```


---


|  |  |  |
| --- | --- | --- |
| **Overview** \| **Package** \| **Class** \| **Use** \| **Tree** \| **Deprecated** \| **Index** \| **Help** | |  |
| **PREV CLASS**   **NEXT CLASS** | **FRAMES**    **NO FRAMES**     **All Classes** |  |
| SUMMARY: NESTED \| FIELD \| CONSTR \| METHOD | DETAIL: FIELD \| CONSTR \| METHOD |  |


---
